# 2123 A clinically relevant rabbit surgical model of pelvic reconstruction to evaluate the immune response to novel surgical materials

**DOI:** 10.1017/cts.2018.38

**Published:** 2018-11-21

**Authors:** Aimon Iftikhar, Alexis Nolfi, Pamela Moalli, Bryan Brown

**Affiliations:** University of Pittsburgh

## Abstract

OBJECTIVES/SPECIFIC AIMS: Pelvic organ prolapse, a disorder in which the muscles of the pelvic floor are weakened over time, affects over a million women each year in the United States. A quarter of these women undergo a reconstructive procedure, increasingly using polypropylene mesh as mechanical reinforcement to the pelvic floor. However, the number of complications such as chronic pain and mesh erosion/exposure in women with vaginal mesh implants were reported at rates as high as 10%–20%. This indicates a limited understanding of the host response to mesh in vaginal tissue and strategies to reduce these complications. Utilizing a novel surgical technique in New Zealand white rabbits, we implant mesh analogously to human implantation and evaluate changes in the immunologic response at early (14 d) and tissue remodeling outcomes at late stages (90 and 180 d) of implantation. The mesh-tissue complex was removed from each rabbit and processed for histological staining as well as immunolabeling of immune cells, such as macrophages. Extracellular matrix protease assays and mechanical integrity of the tissue also evaluate the overall inflammatory response associated with each implant. METHODS/STUDY POPULATION: Commercially available polypropylene mesh was used to investigate the modulation of the immune response. An adapted radio frequency glow discharge method is used to create a stable negative charge on the surface of the mesh, followed by the sequential deposition of polycationic and polyanionic polymers to provide a stable, conformal, nanoscale coating. It is well known macrophages are characterized on a spectrum ranging from a proinflammatory M1 phenotype to an M2 anti-inflammatory phenotype. Interleukin-4, an immunomodulatory cytokine known to promote the M2 phenotype, is incorporated into the coating to be released in a controlled manner upon implantation. Utilizing a novel surgical technique in New Zealand white rabbits, we implant mesh using the “gold standard” abdominal sacrocolpopexy procedure and evaluate changes in the immunologic response at early (14 d) and tissue remodeling outcomes at late stages (90 and 180 d) of implantation. The procedure begins with an initial hysterectomy removing the uterus followed by creating space along the vaginal wall on both sides between the bladder and the rectum. Two 3×10 cm^2^ pieces of mesh are secured along both sides of the vaginal wall. The remaining flaps at the top are then secured to a ligament in the sacral/lumbar space, creating the support to the pelvic organs. Upon closing the incision, a partial thickness defect is made in the abdominal wall, and mesh is implanted inside to repair the abdominal muscle. Both of these implantations of mesh allow for the assessment of the immune response in the pelvic area (relevant for prolapse patients) and in the abdominal area (relevant for translation from hernia repair).The mesh-tissue complex was removed from each rabbit and processed for histological staining as well as immunolabeling of immune cells, such as macrophages. Extracellular matrix protease assays and mechanical integrity of the tissue also evaluate the overall inflammatory response associated with each implant. RESULTS/ANTICIPATED RESULTS: The results of this study show that implants into vaginal tissues elicited an increased host inflammatory response at 14 days as compared with those in the abdominal wall. However, at chronic time points the inflammatory response in the vagina was reduced as compared to that in the abdominal cavity. The present study also demonstrates the scale-up of a previous methodology for a nanoscale coating. We present a nanometer thickness, tunable, and uniform coating capable of releasing bioactive interleukin-4. We evaluated the biological functionality of the coated mesh via bioactivity studies and in vivo implantation. An ideal mesh would provide mechanical support to the pelvic floor while decreasing the inflammatory response and increasing integration with the surrounding native tissue. DISCUSSION/SIGNIFICANCE OF IMPACT: We developed an in vivo model clinically relevant to understanding the early host response to mesh in an anatomically relevant area, the vaginal microenvironment. Previous studies have been conducted in a rodent abdominal defect model while other work has been done in a nonhuman primate vaginal model, but the host response is only observed at later time points (>3 mo). Thus, we developed a rabbit model to investigate early responses and a novel coating to actively working towards improved tissue integration.

